# In Vitro Evaluation of the Healing Potential and Proteomic Study of *Quercus robur* L. Leaf Extracts in Human Keratinocytes

**DOI:** 10.3390/molecules30102152

**Published:** 2025-05-14

**Authors:** Nelson Rojas-Velis, Casimiro Cárdenas-García, Erik Pérez, Jorge R. Toledo, Miguel Ángel Medina, Allisson Astuya-Villalón, Roberto T. Abdala-Díaz

**Affiliations:** 1 Biotoxins Laboratory, Faculty of Natural and Oceanographic Sciences, Center for Oceanographic Research COPAS COASTAL, University of Concepción, Concepción 4070386, Chile; nelsonrojas@udec.cl (N.R.-V.); aastuya@udec.cl (A.A.-V.); 2 Central Research Support Services (SCAI), University of Málaga, Campus de Teatinos s/n, E-29071 Málaga, Spain; ccg@uma.es; 3 Biotechnology and Biopharmaceuticals Laboratory, Pathophysiology Department, Universidad de Concepción, Victor Lamas 1290, P.O. Box 160-C, Concepción 4030000, Chile; erperez@udec.cl (E.P.); jotoledo@udec.cl (J.R.T.); 4 Department of Molecular Biology and Biochemistry, Faculty of Science, Universidad de Málaga, Andalucía Tech, E-29071 Málaga, Spain; medina@uma.es; 5 Department of Ecology, Faculty of Sciences, Institute of Biotechnology and Blue Development (IBYDA), Universidad de Málaga, Campus de Teatinos s/n, E-29071 Málaga, Spain

**Keywords:** *Quercus robur*, wound healing, chronic wounds, plant extracts, antioxidants, proteomics analysis

## Abstract

(1) Background: This study evaluated the potential of an aqueous extract from *Quercus robur* L. leaves for chronic wound healing. Its composition, rich in bioactive compounds (tannins and flavonoids), confers antioxidant and antimicrobial properties. (2) Methods: The toxicity and ability of the extract to enhance cell migration were tested in human keratinocytes (HaCaT cell line). Additionally, a proteomic analysis was performed on treated cells. (3) Results: The extract exhibited low cytotoxicity (IC50 = 943 µg·mL^−1^) compared to other plant extracts. At 5 mg·mL^−1^, it significantly accelerated wound closure at 8 h, surpassing negative control and Reoxcare; however, results were comparable at 12 h. Proteomic analysis identified 117 differentially expressed proteins (21 upregulated, 96 downregulated) involved in essential processes such as cell migration, blood clotting, and cholesterol biosynthesis. Specifically, the extract increased the expression of CYP51A1, LSS, and SQLE, while inhibiting Delta (14)-sterol reductase, key enzymes in cholesterol metabolism, suggesting a potential mechanism for tissue regeneration. (4) Conclusions: The aqueous extract of *Q. robur* leaves shows promise as a natural therapeutic agent for chronic wound healing, potentially aiding tissue regeneration and modulation of cholesterol metabolism.

## 1. Introduction

Wounds are broadly categorized as acute or chronic, differing in their healing mechanisms and closure timelines. They are categorized based on their depth and etiology, with the most challenging to treat being diabetic ulcers, extensive burns, radiation injuries, advanced pressure ulcers, and wounds infected by multidrug-resistant bacteria [1,2,3]. The annual cost of treating chronic wounds in the United States exceeds USD 28 billion, while in Europe it is estimated to consume between 2% and 4% of the total healthcare budget [1]. In Chile, particularly for conditions such as diabetic foot ulcers (DFUs), the cost of treatment varies between USD 102 and USD 3959, while a case requiring amputation can cost between USD 3060 and USD 188,645 [2].

Currently, the market offers a variety of products for the treatment of chronic wounds, such as advanced dressings, antimicrobial agents, and negative pressure therapies [3]. Hydrogel dressings, for example, have proven effective due to their biodegradable, adhesive, and bioactive properties, which can accelerate the healing of chronic wounds [4]. However, despite these options, their efficacy is not universal, and they are often associated with high costs and side effects, highlighting the need to develop new treatments that are effective, safe, and accessible [5]. In this context, plant extracts emerge as a promising alternative due to their diversity of bioactive compounds and historical use in traditional medicine [6]. Plant extracts offer multiple advantages, including low cost, accessibility, and a generally favorable safety profile [7]. Extracts, such as those from green tea (*Camellia sinensis*), reduce LDL cholesterol and improve endothelial function [8], and curcumin from turmeric (*Curcuma longa*) inhibits the proliferation of cancer cells and reduces inflammation in patients with rheumatoid arthritis [9]. Moreover, curcumin has been tested as a dressing-type product in large, hard-to-heal neuro-ischemic ulcers, showing positive results in efficacy and safety [10]. 

Species of *Quercus* spp. (oak) have traditionally been used in medicine and have been the subject of numerous studies due to their many pharmacological properties, including anti-inflammatory, antimicrobial, and antioxidant activity [11,12,13]. These actions are mainly attributed to their high content of tannins and flavonoids, such as quercetin, which inhibit the growth of pathogens and regulate the inflammatory response, thus promoting tissue repair processes [11,12,13,14]. 

In particular, extracts from the bark of *Quercus robur* and *Q. petraea* have demonstrated the ability to protect cells against oxidative stress, reducing damage and promoting skin regeneration [15,16,17,18]. Similarly, in vivo studies with aqueous extracts of Quercus infectoria have shown improvements in wound healing by stimulating angiogenesis and collagen synthesis, with a significant increase in the rate of wound closure and the regulation of factors such as VEGF, which optimize the supply of nutrients and oxygen to the repairing tissue [19,20,21,22,23]. 

To our knowledge, no report in the literature explicitly addresses the molecular mechanisms related to the wound-healing effect of *Q. robur*, highlighting a significant opportunity to investigate its therapeutic potential further. 

Recent studies suggest that plant-derived compounds can modulate key cellular processes, such as migration and proliferation [24]. Given the complex nature of these phenomena, proteomic approaches offer a powerful tool to elucidate these mechanisms by identifying changes in protein expression and functional pathways in response to treatment.

Moreover, it has been reported that quercetin can regulate cholesterol synthesis [25]. This lipid is an essential component of cellular membranes and a precursor of bioactive molecules that modulate cell signaling, inflammatory responses, and cellular differentiation [26,27]. Furthermore, cholesterol can modify proteins in the Hedgehog pathway, particularly the Smoothened protein, which coordinates the proliferation and differentiation processes necessary for proper wound healing [27,28,29]. Under normal physiological conditions, cholesterol metabolism is balanced through biosynthesis, absorption, export, and esterification; however, in pathologies such as diabetes mellitus, this balance is disrupted, favoring hyperlipidemia and adversely affecting wound repair [30,31,32]. Similarly, steroids and angiostatin act on endothelial cells to trigger mitosis, promote cell migration, and stimulate the release of endothelial growth factors [31]. These findings indicate that modulation of cholesterol synthesis could play a crucial role in wound-healing processes; however, no studies have yet comprehensively addressed the role of cholesterol in this process.

In this study, we evaluated the effects of an aqueous extract of *Q. robur* leaves on human keratinocytes (HaCaT cell line) as a model for wound healing. Using proteomic analysis, we aimed to identify differentially expressed proteins and elucidate the biological pathways influenced by the extract. Our findings provide insights into the potential of *Q. robur* as a natural therapeutic agent for chronic wound management.

## 2. Results

The results of this study show the effect of *Q. robur* aqueous leaf extract on cell viability, keratinocyte migration and modulation of key proteins involved in wound healing, and the cholesterol synthesis pathway. The main findings are presented below.

### 2.1. Phytochemical Composition and Antioxidant Activity of the Aqueous Extract from Quercus robur Leaves

The phytochemical characterization of the extract is summarized in Table 1, which reports the determined values for organic nitrogen, minerals, total proteins, and phenolic compounds. The total phenolic compounds showed consistency with antioxidant activity, with a maximum inhibition of 22% at the biggest concentration, as demonstrated by the DPPH assay.

### 2.2. Effect of Quercus robur Extracts on the Cell Viability

**Figure 1:**
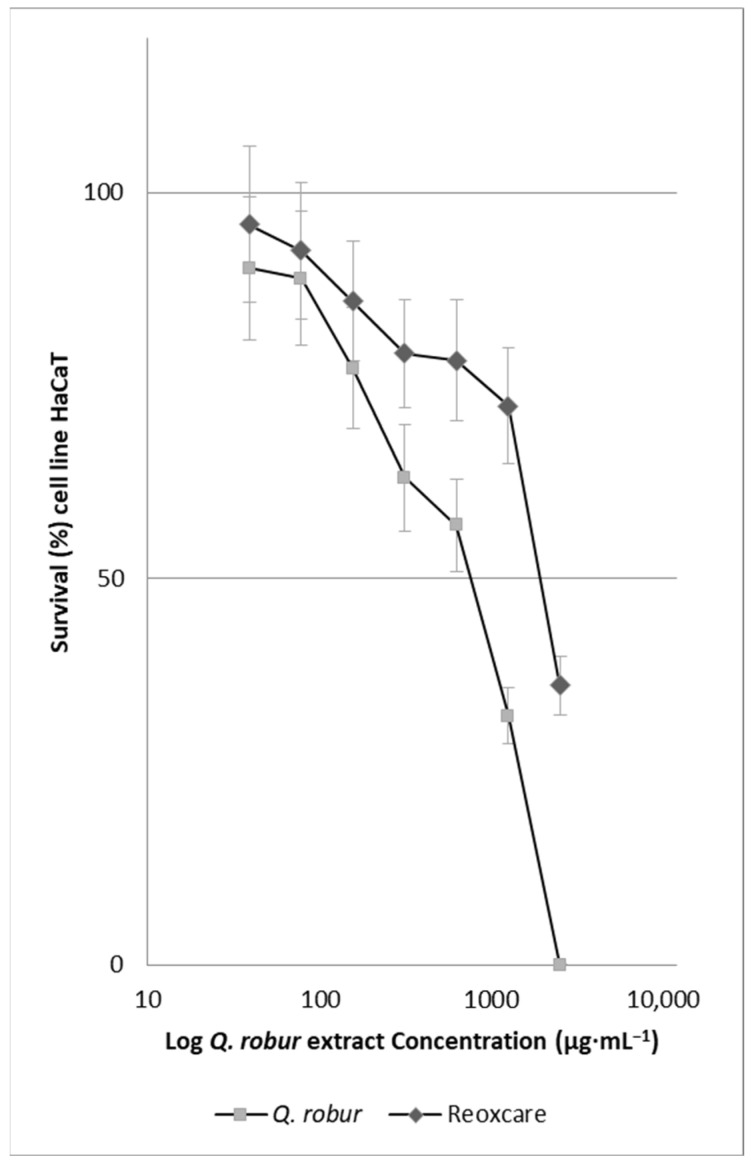
Effect of the extract of *Q. robur* and Reoxcare gel on the viability of HaCaT cells. The graph shows the percentage of cell viability in response to a range of concentrations of the extract (0–2500 µg·mL^−1^; *x*-axis in Log_10_ scale). Error bars indicate the standard deviation of the measurements. The graph shows three experiments performed in different weeks, with three replicates for each one (n = 3, N = 3).

Figure 1 shows that, as the concentration of the extract increases, cell survival decreases, indicating a negative linear relationship. The extract concentration that inhibits 50% of the absorbance of MTT converted to formazan by the respiratory activity of cells (IC50), which estimates the number of living cells in the wells, was 943 µg·mL^−1^ for the *Q. robur* extract. For the positive control, Reoxcare gel, formulated with turmeric extract and used as a reference in this study, the estimated IC50 value was 1967 µg·mL^−1^. 

### 2.3. Effects of Quercus robur Extracts on Cell Migration

**Figure 2:**
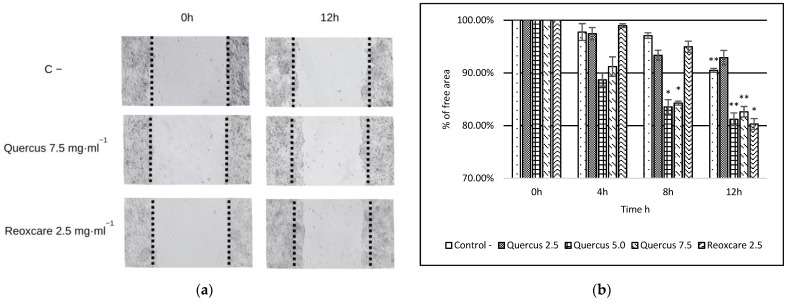
The effects of *Quercus robur* L. and Reoxcare on the migratory potential of the immortalized human keratinocyte cell line (HaCaT) were assessed using the wound-healing assay. (**a**) shows representative images of the negative control (C −) and cells treated with various concentrations of *Q. robur* extract and Reoxcare at 0 and 12 h (with 4 and 8 h not shown). (**b**) presents the relative quantification (in percentages) of the cell-free areas in samples treated with increasing concentrations of *Q. robur* extract and a fixed concentration of Reoxcare over the same time points. Statistical significance compared to the negative control was determined using a two-sided unpaired Student’s *t*-test: * *p* < 0.05; ** *p* < 0.005.

In this study, we also assessed the effects of treatment with *Q. robur* extract (2.5 mg·mL^−1^, 5.0 mg·mL^−1^, and 7.5 mg·mL^−1^) and the product Reoxcare (2.5 mg·mL^−1^) on cell migration using a wound-healing assay conducted on HaCaT cells evaluated at 0, 4, 8, and 12 h; the most significant results are presented in Figure 2. Cell migration was expressed as a percentage of cell-free area. Initially, all samples had a 100% cell-free area, establishing a uniform baseline for comparison. 

At 4 and 8 h, a moderate reduction in overall cell-free area was observed across all treatments. At 12 h, the trend toward progressive gap closure became even more apparent. The control group showed a cell-free area of 90.49%, while the *Q. robur* treatments at 5.0 and 7.5 mg·mL^−1^ achieved values of 81.21% and 82.64%, respectively. For Reoxcare at 2.5 mg·mL^−1^, the cell-free area decreased to 80.33%, indicating more pronounced cell migration. Differences between groups were assessed using Student’s *t*-test for independent samples. The reduction in cell-free area from 0 h to 12 h in the control was significant, indicating a slight basal migratory effect. Furthermore, treatment with *Q. robur* at 5.0 mg·mL^−1^ resulted in significant differences at both 8 h and 12 h. Similarly, *Q. robur* at 7.5 mg·mL^−1^ exhibited significant differences at 8 h and 12 h. Finally, Reoxcare at 2.5 mg·mL^−1^ demonstrated a significant reduction in cell-free area at 12 h. Overall, these results suggest that gap closure occurs progressively in all groups but is accelerated in cells treated with higher concentrations of *Q. robur* and Reoxcare. The treatments induced cell migration are statistically significant when compared to the negative control, suggesting that both compounds may enhance the wound-healing process.

### 2.4. Proteomic Analysis

#### 2.4.1. Effects of Aqueous Extract of *Quercus robur* on the Proteomic Profile of Human Keratinocytes

**Figure 3:**
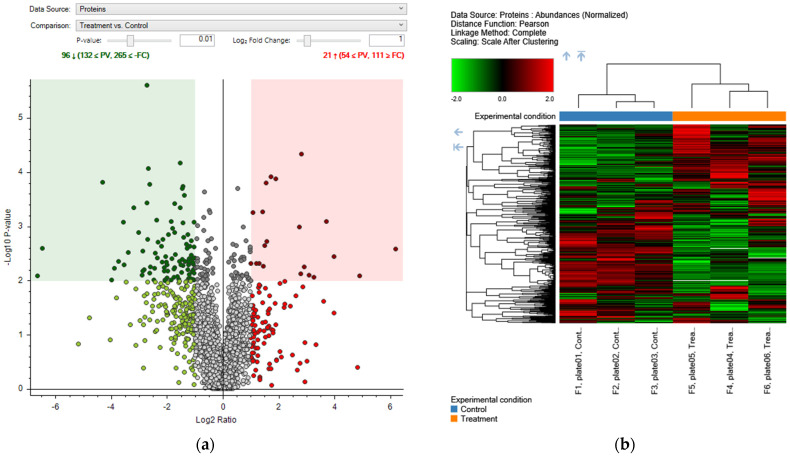
(**a**) Volcano plot displaying the differentially expressed proteins (DEPs) in HaCaT cells treated with *Q. robur* extract compared to the untreated control group. The *X*-axis represents the base-2 logarithm of the expression fold change (Log_2_ Ratio), and the *Y*-axis represents the negative base-10 logarithm of the *p*-value (–log_10_ *p*-value). Red dots indicate upregulated DEPs, while green dots represent downregulated DEPs. Gray dots denote proteins whose expression did not differ significantly between the groups. The threshold for statistical significance was set at a *p*-value < 0.01. In total, 96 proteins showed significant expression changes, with 21 upregulated and 75 downregulated. (**b**) Heatmap showing the DEPs in HaCaT cells treated with *Q. robur* extract compared to the control group (Ctrl). Protein abundance data were normalized and scaled after clustering. Pearson’s correlation was used as the distance function, and complete linkage was employed as the clustering method. Each row represents a protein, and each column represents an experimental sample. Proteins are hierarchically clustered based on their expression patterns. Colors indicate relative expression levels: green for low expression, black for medium expression, and red for high expression. Control samples are labeled as “Cont.” and treated samples as “Trea”. The dendrograms at the top and left illustrate the similar relationships among samples and proteins, respectively. The arrows mark the cut points in the column (top) and row (left) dendrograms delineating the two main clusters of samples (Control vs. Treatment) and proteins.

Proteomic analysis revealed significant changes in protein expression following treatment with the *Q. robur* extract. A total of 117 differentially expressed proteins (DEPs) were identified, comprising 21 upregulated and 96 downregulated proteins. These findings are illustrated in the volcano plot (Figure 3a), showing protein expression changes (Log_2_ Ratio) against statistical significance (−log_10_ *p*-value). The significance threshold was set at *p* < 0.01. Additionally, a heatmap (Figure 3b) highlights distinct expression patterns between treated and control groups, allowing visualization of the hierarchical clustering of DEPs.

#### 2.4.2. Protein–Protein Interaction (PPI) Network Analysis

**Figure 4:**
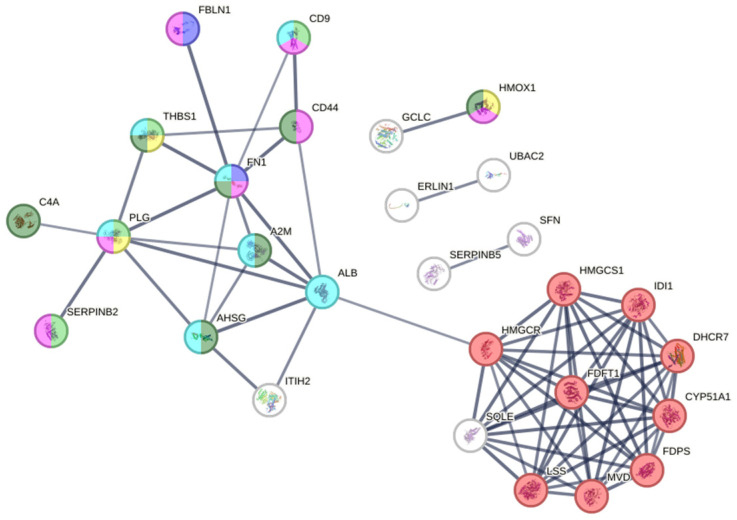
Visualization of the protein–protein interaction (PPI) network using STRING. The network consists of 50 nodes and 71 edges, with an expected number of edges being 8, and a PPI enrichment *p*-value of <1.0 × 10^−16^, indicating significantly higher connectivity than expected. The average node degree is 2.84, and the average local clustering coefficient is 0.495. The network reveals two main clusters: a dense one (in red) associated with platelet degranulation and a less dense one (blue/green) related to processes such as blood coagulation and inflammatory response. These clusters reflect the identified enriched biological functions and pathways.

STRING analysis resulted in a PPI network consisting of 50 nodes and 71 edges, with connectivity significantly exceeding random expectations. (*p* < 1 × 10^−16^). This analysis identified key proteins involved in biological processes, including cell migration, blood coagulation, and cholesterol biosynthesis. Figure 4 provides a visual representation of the network, where nodes with more connections indicate central proteins in metabolic and signaling pathways.

A notable finding is the overexpression of proteins involved in cholesterol biosynthesis. Figure 4 shows a significant cluster of proteins associated with this pathway, highlighting key enzymes such as CYP51A1, DHCR7, FDFT1, LBR, LSS, and SQLE. This overexpression suggests that *Q. robur* extract may modulate cholesterol synthesis, a critical process for cellular regeneration and wound healing [26,27,28].

#### 2.4.3. Functional Enrichment Analysis

Functional enrichment analysis identified significant Gene Ontology (GO) terms related to processes such as cell migration regulation, adhesion, and wound healing (Table 2). Additionally, Reactome pathway enrichment analysis highlighted key metabolic pathways, such as platelet degranulation, with a *p*-value of 1.44 × 10^−5^ (Table 3). These findings emphasize the role of *Q. robur* extract in modulating essential biological processes involved in skin regeneration.

#### 2.4.4. Steroid Biosynthesis Pathway

**Figure 5:**
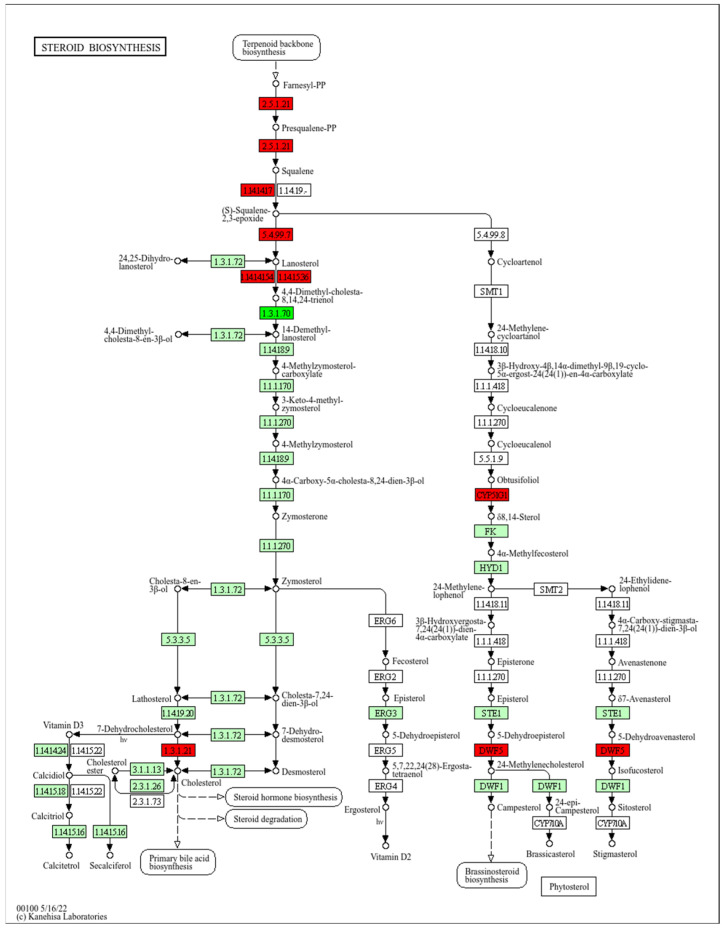
Steroid biosynthesis pathway in *Homo sapiens* (hsa00100) showing overexpressed (red) and underexpressed proteins (bright green) when cells were treated with aqueous extract of *Q. robur*. The image was obtained by a valid license from the KEGG module within Proteome Discoverer software 2.5 (Thermo Fisher Scientific, Waltham, MA, USA).

The analysis of the steroid biosynthesis pathway in *Homo sapiens* is presented in Figure 5, highlighting the key enzymes involved in this process. The pathway begins with the biosynthesis of the terpenoid backbone, where farnesyl pyrophosphate (Farnesyl-PP) is converted into squalene by the enzyme farnesyl-diphosphate farnesyltransferase 1 (FDFT1, hsa:2222). Subsequently, squalene is epoxidized by squalene epoxidase (SQLE, hsa:6713) to form 2,3-oxidosqualene, which is converted into lanosterol via lanosterol synthase (LSS, hsa:4047).

The figure also highlights the involvement of key enzymes such as CYP51A1 (hsa:1595), involved in the demethylation of lanosterol, and DHCR7 (hsa:1717), which reduces 7-dehydrocholesterol to cholesterol. Additionally, the lamin B receptor (LBR, hsa:3930) plays a role in the conversion of zymosterol to cholesta-7,24-dien-3β-ol. These enzymatic steps are crucial for the biosynthesis of cholesterol and other steroids, which are essential for the structure and function of cellular membranes and the production of steroid hormones. However, the stability or fluidity of the membrane after treatment with the extract was not directly assessed in this work.

## 3. Discussion

The search for new therapeutic agents to treat chronic wounds is a crucial area of research, driven by the increasing prevalence of these conditions and the limitations of current treatments. In this context, the use of plant extracts with bioactive properties has become a focus of interest, thanks to their potential to promote healing, reduce inflammation, and prevent infections [22,32,33].

Results obtained from *Q. robur* leaves indicated that the content of organic nitrogen (N), phosphorus (P), and protein fell within the ranges reported for both this species and other members of the genus [34,35]. The total phenolic content measured 3.2 mg GAE·g^−1^ DW, and the antioxidant activity (maximum DPPH inhibition of 22%) was moderate compared to previous studies. Reported phenolic contents for other *Quercus* species ranged between 11.20 and 35.47 mg GAE·g^−1^ DW [18,36,37], demonstrating significantly higher antioxidant activity and reaching up to 81.14% (*Q. sartorii*) and 82.60% (*Q. rysophylla*) inhibition in methanolic extracts [38]. These differences are attributed to the extraction method and the plant material used. While most reported studies utilize dried bark and organic solvents at elevated temperatures, our protocol employs fresh leaves and aqueous extraction at room temperature. We did not identify studies based on aqueous extracts of *Q. robur* leaves. This approach simulates a simple, sustainable, reproducible, scalable, and low-cost preparation method that could facilitate the development of a *Q. robur*-based wound-healing product.

The cytotoxicity analysis of the aqueous extract of *Q. robur*, conducted using the MTT assay, revealed an IC50 of 943 µg·mL^−1^ [39], aligning with findings in keratinocytes and fibroblasts: Hong et al. observed ~90% viability in HaCaT cells exposed to 100 µg·mL^−1^ of *Q. acuta* aqueous extract, and Wunnoo et al. reported ~70% viability in fibroblasts treated with 200 µg·mL^−1^ of *Q. infectoria* ethanolic extract [39,40]. Taken together, these data indicate a low level of cytotoxicity for the *Q. robur* extract under the conditions tested.

Previous studies have demonstrated that extracts from various *Quercus* species, including *Q. coccifera*, can promote the proliferation of 3T3 fibroblasts and affect TNF-α production in macrophages [22]. These effects relate to cellular processes involved in tissue repair; however, enhanced cell proliferation in vitro does not necessarily signify regenerative activity in tissues. Although the tested concentrations provide valuable insight into the cytotoxicity thresholds of *Q. robur*, we recognize that, by exceeding physiologically or clinically attainable levels, they may compromise the extract’s practical efficacy and thus warrant validation in more representative models. 

Compared to the commercial product Reoxcare gel, the *Q. robur* extract exhibited a lower IC50 (1967 µg·mL^−1^ for Reoxcare), indicating the higher cytotoxicity of the *Q. robur* extract under the evaluated conditions. However, the gel formulation includes additional components that may influence cellular responses, which should be considered when interpreting these values. In the wound-healing assay, the *Q. robur* extract promoted cell migration after 8 h of treatment, showing significant differences from the negative control and Reoxcare. After 12 h, the effect was like that of Reoxcare, with 80% of the wound remaining unclosed. To better assess its impact on tissue regeneration, further studies could extend the experiment duration or include cell division inhibitors, such as mitomycin C, to differentiate migration effects from proliferation-related effects [41]. The *Q. robur* extract contains phenolic compounds, including gallic acid, catechin, and epicatechin, which are recognized for their antioxidant and anti-inflammatory properties. In cellular models, gallic acid benefits cell migration and wound healing, even under oxidative stress or hyperglycemia [14]. While these mechanisms may play a role in the observed effects, further studies are needed to validate this hypothesis.

The integration of these findings suggests that the *Q. robur* extract, with its high concentration of phenolic compounds, may promote a pro-migratory effect on keratinocytes by modulating oxidative stress and inflammation. This effect is particularly relevant in chronic wounds, where early cell migration is crucial in tissue recovery. 

The proteomic analysis revealed significant changes in cholesterol metabolism, including the overexpression of proteins such as CYP51A1 and DHCR7, which play a crucial role in cholesterol biosynthesis. Previous studies have demonstrated that inhibiting the mevalonate pathway with statins such as pravastatin can decrease keratinocyte proliferation and induce cell cycle arrest, whereas cholesterol supplementation can partially reverse this effect. These findings underscore the importance of cholesterol in regulating cell cycle proteins through pathways like AKT and ERK [42]. While the noted upregulation of CYP51A1 and DHCR7 hints at a potential connection between cholesterol metabolism and keratinocyte behavior, additional studies are necessary to establish whether this pathway has a direct role in the effects of the *Q. robur* extract on wound healing.

### 3.1. Role of Cholesterol Synthesis in Wound Healing

Cholesterol synthesis is vital for skin wound healing, especially in forming and maintaining the epidermal barrier, which consists of keratinocytes and an extracellular lipid matrix, including cholesterol. Studies indicate that cholesterol plays a role in keratinocyte proliferation and differentiation, both important for skin regeneration [42]. Enzymes in the cholesterol synthesis pathway, such as CYP51A1, LSS, and SQLE, contribute to the production of cholesterol and isoprenoids, which are critical for tissue regeneration. Our study’s findings of enzyme overexpression suggest that *Q. robur* extract may influence cholesterol synthesis, potentially aiding the healing process [43,44,45,46]. Proteomic analysis revealed a reduction in Delta (14)-sterol reductase (LBR) levels in treated samples compared to controls. Although LBR may not be fully inhibited, its reduced detection implies potential modulation by *Q. robur* extract. Since LBR is involved in cholesterol biosynthesis, these findings may indicate an indirect effect of the extract on cholesterol-related pathways in wound healing. 

Although our study focused primarily on the modulation of cholesterol biosynthesis as a potential mechanism of action, proteomic analysis revealed additional differentially expressed proteins involved in key biological processes such as cell migration, blood coagulation, and inflammatory response (Appendix A, Table A1). These findings suggest that *Quercus robur* leaf extract may exert a multifactorial influence on wound healing, a hypothesis that warrants further investigation.

### 3.2. Suggested Mechanism of Action

The bioactive compounds present in the *Q. robur* extract include tannins (such as hydrolyzable tannins, gallotannins, and ellagitannins), flavonoids (kaempferol, quercetin, and isorhamnetin), simple phenolic compounds (gallic acid and ellagic acid), and triterpenoids like lupeol. Tannins possess astringent properties that contract tissues and stop hemorrhages, which is crucial in the initial phase of healing. Flavonoids are known for their antioxidant and anti-inflammatory effects, helping to protect damaged tissues and promote healing. Similarly, simple phenolic compounds like gallic and ellagic acids exhibit antioxidant and anti-inflammatory properties, potentially benefiting the wound-healing process. Lupeol, a triterpenoid isolated from various *Quercus* species, inhibits COX-1 and COX-2 enzymes involved in inflammation, contributing to anti-inflammatory and cytotoxic effects [13,47]. The combination of these compounds could enhance cell proliferation and migration, reduce oxidative stress at the wound site, and modulate essential metabolic pathways, explaining the extract’s efficacy in accelerating the repair process.

### 3.3. Future Directions

Being in vitro research, the results may not fully replicate the complex biological environment of chronic wounds in vivo. Therefore, animal studies are recommended to validate the efficacy and safety of *Q. robur* extract under conditions more representative of the human organism. Moreover, delving into the molecular mechanisms through detailed studies of the signaling pathways and protein interactions is crucial for a more complete understanding of how the extract influences healing. Evaluating the impact on other key cell types, such as fibroblasts and endothelial cells, could expand knowledge about its therapeutic potential.

## 4. Materials and Methods

### 4.1. Biological Materials and Extracts from Quercus robur

*Quercus robur* L. is a tree belonging to the family Fagaceae and was described by Charles Linnaeus and published in 1753 [48]. In this study, an aqueous extract was obtained from *Q. robur* leaves collected in January 2023 in the San Carlos commune, Bío Bío region, Chile. The selected leaves were fully developed and in good condition; we avoided those with signs of disease, insect damage, mold, or discoloration. For the extraction, 27 grams of *Q. robur* leaves were placed in a flask containing 600 mL of distilled water. The leaves were blended and then subjected to continuous stirring for 24 h at room temperature. Subsequently, the mixture was distributed into 50 mL tubes and centrifuged at 1107× *g* for 15 min. The obtained supernatant was collected and lyophilized to obtain the final aqueous extract.

Reoxcare gel is a commercial product, a hydrogel with antioxidant ingredients, mainly turmeric extract, which is used to treat skin lesions. For this study, it was used as a control in the toxicity and cell migration tests [49].

### 4.2. Analytical Methods for the Characterization of Compounds and Antioxidant Activity in Quercus robur Leaves

The determination of total nitrogen was carried out using the Kjeldahl method [50]. This method entails digesting the dried sample with concentrated sulfuric acid and catalysts to convert organic nitrogen into ammonium ions. These ions are then distilled and quantified by molecular absorption spectrophotometry according to the specifications of Standard Methods 4500-NH_3_ (n = 3) [51]. For the determination of total phosphorus, the procedure described by Murphy and Riley was applied [52], which involves thorough digestion using oxidizing reagents and concentrated acids to transform all forms of phosphorus into phosphate, followed by colorimetric quantification through the formation of a blue complex of reduced molybdate–phosphomolybdate with ascorbic acid, in accordance with Standard Methods 4500-P (n = 3) [53]. Total protein was calculated by multiplying the total nitrogen value by the conversion factor 6.25 [54].

The total phenolic content of fresh *Q. robur* leaves was determined from samples pulverized in a mortar with liquid nitrogen and extracted overnight in centrifuge tubes with 2.5 mL of 80% (*v*/*v*) methanol (n = 3). The mixture was centrifuged at 19,000× *g* for 15 min, and the supernatant was collected, as described by Abdala Díaz et al. [55]. Total phenolic compounds were determined using gallic acid (Sigma-Aldrich, St. Louis, MO, USA) as a standard, and the absorbance was measured at 760 nm using a UV-visible spectrophotometer (SHIMADZU UV MINI-1240, Shimadzu, Duisburg, Germany) [56]. Phenolic concentration was expressed as gallic acid equivalent (GAE) per gram dry weight (DW).

The antioxidant and radical scavenging activities of biomass extracts were evaluated by the 2,2-diphenyl-1-picrylhydrazil (DPPH) free radical method of Brand-Williams et al., 1995 [57]. The biomass extract was obtained from 20 mg of dry weight of the crushed *Q. robur* leaves, and 2.5 mL of MeOH at 80% was added. Dilutions were made to achieve final concentrations in the cuvettes of 200, 100, 50, 25, 12.5, and 6.25 µg·mL^−1^. DPPH (Sigma-Aldrich, St. Louis, MO, USA) (1000 µL) was added to the cuvettes, along with 80% MeOH and 200 µL of the sample. The initial absorbance was measured at 517 nm. The samples were incubated for 30 min, and the absorbance was recorded again at 517 nm, using 80% MeOH (Sigma-Aldrich, St. Louis, MO, USA) as the solvent. The absorbance was then transformed into a percentage inhibition against 80% MeOH. The percentage of the antioxidant activity was calculated according to Equation (1):

AA% = [(Abs_0_ − Abs_1_) Abs_0_] × 100 (1)

where A_0_ is the absorbance at time 0 min, and A_1_ is the absorbance at the end of the reaction (30 min) at 517 nm. A calibration curve was created using different concentrations of Trolox^®^ from a stock solution of 1.268 mM Trolox^®^. Dilutions were obtained at concentrations from 0 to 6 µM. All determinations were performed in triplicate (n = 3) [58]. The antioxidant capacity was expressed as % of antioxidant activity.

### 4.3. Cell Culture and Treatments

An immortalized human keratinocyte cell line, HaCaT (ATCC, Manassas, VA, USA), was used and cultured in RPMI-1640 medium (BioWhittaker, Walkersville, MD, USA, ref. BE12-167F) supplemented with 10% fetal bovine serum (FBS, Biowest, Lakewood Ranch, FL, USA, ref. S1810-500), 1% penicillin-streptomycin 100× solution (Capricorn Scientific, Ebsdorfergrund, Germany, ref. PS-B), and 0.5% amphotericin B (Biowest, ref. L0009-100). The cells were maintained subconfluent at 37 °C in a humidified atmosphere containing 5% CO_2_ [59].

### 4.4. Cytotoxicity Assays (MTT Assays)

The cytotoxic effect of *Quercus robur* extract and Reoxcare on HaCaT cell lines was evaluated using the MTT assay. Cells were seeded into 96-well plates at a density of 1 × 10^4^ cells per well and allowed to adhere overnight. Treatments were applied at increasing concentrations (0 to 2.5 mg·mL^−1^), with each extract dissolved in RPMI medium prior to dilution. Cells were incubated with the treatments at 37 °C in a humidified atmosphere with 5% CO_2_ for 72 h. Control wells contained only RPMI medium without any test substances.

After incubation, wells were gently washed once with phosphate-buffered saline (PBS 1×) to remove residual treatment. Then, 100 µL of MTT solution was added to each well and incubated for 4 h at 37 °C. Subsequently, 150 µL of acidified isopropanol was added to each well to solubilize the formazan crystals. The plate was gently shaken, and absorbance was measured at 570 nm using a microplate reader.

The assay was performed in three independent experiments (N = 3). Cytotoxicity was calculated and expressed as the 50% inhibitory concentration (IC50). The methodology was adapted from Abdala Díaz et al. [60], with modifications as described above.

### 4.5. Cell Migration Assay

According to the manufacturer’s instructions, the migration assay was completed using the Culture-Insert 2 well migration assay kit (Ibidi) [61]. HaCaT cells were seeded into the two-well plates at a density of 3 × 10^5^ cells·mL^−1^, using 70 μL per well, and cultured in standard medium until they reached confluence, approximately 2 days in normal culture medium RPMI supplemented with 10% FBS and 1% penicillin-streptomycin, in a humidified atmosphere with 5% CO_2_ at 37 °C (N = 3). The negative control consisted of untreated cells, while the positive control was Reoxcare gel. After removing the insert, the cells were treated with different concentrations of aqueous extract of *Q. robur* leaves, and HaCaT migration activity in the space between the insert wells was visualized at 0, 4, 8, and 12 h by inverted microscopy (Olympus IX71, Olympus, Tokyo, Japan). During treatment, normal culture medium without FBS was used. With the images, the area of gap closure was calculated using Adobe Photoshop area selection and calculation tools. Statistical significance was determined by a two-sided unpaired Student’s *t*-test. 

### 4.6. Proteomic Analysis 

A 6-well plate was prepared, with 3 wells designated for treatment with *Q. robur* extract (5 mg·mL^−1^) under identical conditions to those of the cell migration and proliferation assay and the other 3 wells used as untreated controls. The cells were cultured for 24 h and then washed with phosphate-buffered saline (PBS) and stored at −80 °C. They were solubilized with 500 μL of RIPA buffer supplemented with universal nuclease and sonicated for five minutes in an ice bath (Ultrasons, J.P. Selecta S.A., Barcelona, Spain). After centrifugation at 14,000× *g*, proteins were purified using a precipitation kit (Clean-Up Kit, GE Healthcare, Tokyo, Japan), and the precipitates were dissolved in Milli-Q water. Protein concentration was normalized to 1 μg·μL^−1^ using a BCA colorimetric assay.

Subsequently, the proteins were mixed with acrylamide monomers and polymerized into polyacrylamide gels. Reduction steps with DTT and alkylation with iodoacetamide were performed. In-gel enzymatic digestion was carried out using trypsin, followed by the extraction of the resulting peptides. These peptides were analyzed by liquid chromatography coupled to mass spectrometry (LC-MS).

For the proteomic analysis, samples were injected into an Easy-nLC 1200 UHPLC system coupled to a Q-Exactive HF-X hybrid quadrupole-Orbitrap mass spectrometer (Thermo Fisher Scientific, Waltham, MA, USA). Peptides were separated on an analytical column and ionized, being classified based on their mass-to-charge ratio (*m*/*z*). The obtained mass spectra were compared with the human protein database from SwissProt using Proteome Discoverer 2.5 software with the Sequest HT search engine (Thermo Fisher Scientific, Waltham, MA, USA). The false discovery rate (FDR) was determined using Percolator, accepting only proteins with at least two peptide sequences. Protein expression quantification was performed using the Minora feature of Proteome Discoverer 2.5, evaluating differences in expression between the treatment and control groups using ANOVA and determining significance with a *p*-value less than 0.05 [62]. 

## 5. Conclusions

In conclusion, this study highlights the significant wound-healing potential of *Q. robur* extract, supported by its effect on cell migration in keratinocytes. It is known that oak bark extracts, specifically, have been used in various traditional medicinal applications due to their astringent, anti-inflammatory, and antioxidant properties. These properties make the *Quercus* extract a valuable addition to the treatment of skin wounds. Additionally, the activation of key proteins involved in cholesterol synthesis was demonstrated in this study. These findings pave the way for future clinical research and the development of new plant-based therapeutic strategies for treating chronic wounds and high cholesterol.

However, it should be noted that the concentrations tested in this study may exceed physiologically or clinically relevant levels, which could limit the translational applicability of the extract and underline the need for validation in more representative in vivo models.

## Figures and Tables

**Table 1:** Leaf extract profile of *Q. robur*; data are expressed in % and mg·g^−1^ DW, including their standard deviation.

Compound/Element	Amount
Total Nitrogen (N)	2.38 ± 0.2%
Phosphorus (P)	0.09 ± 0.001%
Total protein	14.87 ± 0.9%
Total phenolic	3.20 ± 0.15 mg·g^−1^ DW

**Table 2:** Biological process (Gene Ontology). This table lists the Gene Ontology (GO) biological process terms that are enriched in the protein–protein interaction (PPI) network. GO terms provide functional descriptions of the biological processes involving the proteins in the network. Strength represents the ratio between the proportion of proteins of the GO term in the network and the expected proportion in the entire genome.

GO-Term	Description	Count in Network	Strength	False Discovery Rate (FDR)
GO:0010647	Regulation of substrate-dependent cell migration, cell adhesion	2	2.42	0.021
GO:0006695	Cholesterol biosynthetic process	2	1.97	7.95 × 10^−4^
GO:0007596	Blood coagulation	9	1.52	3.12 × 10^−4^
GO:0002040	Positive regulation of endothelial cell migration	7	0.77	0.0099
GO:0022617	Wound healing	8	0.77	0.028
GO:0006954	Inflammatory response	8	0.77	0.0077

**Table 3:** “Reactome Pathways”. This table lists the Reactome pathways enriched in the protein–protein interaction (PPI) network. Reactome pathways provide detailed descriptions of the metabolic and signaling routes in which the network proteins are involved.

Pathway	Description	Count in Network	Strength	False Discovery Rate (FDR)
R-HSA-114608	Platelet degranulation	7	1.34	1.44 × 10^−5^

## Data Availability

The original contributions presented in this study are included in the article. Further inquiries can be directed to the corresponding author.

## Abbreviations

The following abbreviations are used in this manuscript:

MTT	(3-[4,5-dimethylthiazol-2-yl]-2,5 diphenyl tetrazolium bromide) assay
GAE	Gallic acid equivalent
DPPH	2,2-diphenyl-1-picrylhydrazyl
LC-MS	Liquid chromatography-mass spectrometry
UHPLC	Ultra-high-performance liquid chromatography
FDR	False discovery rate
DEPs	Differentially expressed proteins
PPI	Protein–protein interaction
GO	Gene Ontology
DTT	Dithiothreitol
BCA	Bicinchoninic acid assay

## Appendix A. Proteins

### Overview of Important Differentially Expressed

**Table A1:** This table summarizes the main differentially expressed proteins (DEPs) identified through proteomic analysis of human keratinocytes treated with *Quercus robur* L. leaf extract. The table includes the gene symbol, full protein name, fold change (FC) values with two decimal precision, and *p*-values with four decimal precision. Molecular functions are provided based on GO annotations.

Gene Symbol	Protein Name	Fold Change (FC)	FC *p*-Value	Molecular Function
AHSG	Alpha-2-HS-glycoprotein [OS = *Homo sapiens*]	72.99	0.0026	enzyme regulator activity
FBLN1	Fibulin-1 [OS = *Homo sapiens*]	30.18	0.0081	signal transduction activity or receptor binding; enzyme regulator activity; extracellular structural activity; other molecular function
PLG	Plasminogen [OS = *Homo sapiens*]	15.82	0.0037	signal transduction activity or receptor binding; other molecular function
ALB	Albumin [OS = *Homo sapiens*]	13.17	0.0008	nucleic acid binding activity; other molecular function
IGFBP2	Insulin-like growth factor-binding protein 2 [OS = *Homo sapiens*]	9.59	0.0087	signal transduction activity or receptor binding; other molecular function
IDI1	Isopentenyl-diphosphate Delta-isomerase 1 [OS = *Homo sapiens*]	8.5	0.008	other molecular function
HMOX1	Heme oxygenase 1 [OS = *Homo sapiens*]	7.62	0.0056	other molecular function
HMGCS1	Hydroxymethylglutaryl-CoA synthase, cytoplasmic [OS = *Homo sapiens*]	7.08	0.0	other molecular function
MVD	Diphosphomevalonate decarboxylase [OS = *Homo sapiens*]	6.92	0.0076	other molecular function
FDFT1	Squalene synthase [OS = *Homo sapiens*]	6.7	0.001	other molecular function
CPD	Carboxypeptidase D [OS = *Homo sapiens*]	3.71	0.0001	other molecular function
GALE	UDP-glucose 4-epimerase [OS = *Homo sapiens*]	3.3	0.0001	other molecular function
GNAI1	Guanine nucleotide-binding protein G(i) subunit alpha-1 [OS = *Homo sapiens*]	2.97	0.0019	signal transduction activity or receptor binding; other molecular function
DHCR7	7-dehydrocholesterol reductase [OS = *Homo sapiens*]	2.92	0.0002	other molecular function
SERPINB5	Serpin B5 [OS = *Homo sapiens*]	2.82	0.0023	enzyme regulator activity
POLR2A	DNA-directed RNA polymerase II subunit RPB1 [OS = *Homo sapiens*]	2.72	0.0054	nucleic acid binding activity; other molecular function
EED	Polycomb protein EED [OS = *Homo sapiens*]	2.7	0.0005	enzyme regulator activity; other molecular function
ROCK2	Rho-associated protein kinase 2 [OS = *Homo sapiens*]	2.48	0.0048	cytoskeletal activity; kinase activity; other molecular function
GRN	Progranulin [OS = *Homo sapiens*]	2.3	0.0048	signal transduction activity or receptor binding; other molecular function
SEC23IP	SEC23-interacting protein [OS = *Homo sapiens*]	2.12	0.0006	other molecular function
ANXA11	Annexin A11 [OS = *Homo sapiens*]	2.02	0.0049	other molecular function
PKP3	Plakophilin-3 [OS = *Homo sapiens*]	0.5	0.0059	other molecular function
DDX54	ATP-dependent RNA helicase DDX54 [OS = *Homo sapiens*]	0.49	0.0038	signal transduction activity or receptor binding; other molecular function
THUMPD1	THUMP domain-containing protein 1 [OS = *Homo sapiens*]	0.49	0.0074	nan
CSNK1A1	Casein kinase I isoform alpha [OS = *Homo sapiens*]	0.49	0.0008	kinase activity; other molecular function
UHRF1	E3 ubiquitin-protein ligase UHRF1 [OS = *Homo sapiens*]	0.49	0.0098	nucleic acid binding activity; other molecular function
DHX16	Pre-mRNA-splicing factor ATP-dependent RNA helicase DHX16 [OS = *Homo sapiens*]	0.49	0.0024	other molecular function
SARNP	SAP domain-containing ribonucleoprotein [OS = *Homo sapiens*]	0.48	0.0063	nucleic acid binding activity; other molecular function
DNMT1	DNA (cytosine-5)-methyltransferase 1 [OS = *Homo sapiens*]	0.48	0.0049	nucleic acid binding activity; other molecular function
DPF2	Zinc finger protein ubi-d4 [OS = *Homo sapiens*]	0.48	0.0039	other molecular function
GNL2	Nucleolar GTP-binding protein 2 [OS = *Homo sapiens*]	0.47	0.0095	other molecular function
TNKS1BP1	182 kDa tankyrase-1-binding protein [OS = *Homo sapiens*]	0.47	0.0046	other molecular function
MAP2K4	Dual specificity mitogen-activated protein kinase kinase 4 [OS = *Homo sapiens*]	0.47	0.0068	kinase activity; other molecular function
CTCF	Transcriptional repressor CTCF [OS = *Homo sapiens*]	0.46	0.0047	nucleic acid binding activity; other molecular function
TMPO	Lamina-associated polypeptide 2, isoforms beta/gamma [OS = *Homo sapiens*]	0.46	0.0018	nucleic acid binding activity; other molecular function
SRSF7	Serine/arginine-rich splicing factor 7 [OS = *Homo sapiens*]	0.46	0.0034	nucleic acid binding activity; other molecular function
LIMA1	LIM domain and actin-binding protein 1 [OS = *Homo sapiens*]	0.45	0.0042	cytoskeletal activity; other molecular function
PPP1R18	Phostensin [OS = *Homo sapiens*]	0.45	0.0091	cytoskeletal activity; other molecular function
ATXN2L	Ataxin-2-like protein [OS = *Homo sapiens*]	0.44	0.0097	nucleic acid binding activity
MARK2	Serine/threonine-protein kinase MARK2 [OS = *Homo sapiens*]	0.44	0.0023	enzyme regulator activity; cytoskeletal activity; kinase activity; other molecular function
EPB41L2	Band 4.1-like protein 2 [OS = *Homo sapiens*]	0.44	0.0044	cytoskeletal activity; other molecular function
MT1H	Metallothionein-1H [OS = *Homo sapiens*]	0.44	0.0039	other molecular function
RBM10	RNA-binding protein 10 [OS = *Homo sapiens*]	0.43	0.0077	nucleic acid binding activity; other molecular function
PELP1	Proline-, glutamic acid- and leucine-rich protein 1 [OS = *Homo sapiens*]	0.43	0.0092	other molecular function
YTHDF3	YTH domain-containing family protein 3 [OS = *Homo sapiens*]	0.43	0.0051	translation activity; nucleic acid binding activity
KPNA2	Importin subunit alpha-1 [OS = *Homo sapiens*]	0.42	0.0063	other molecular function
PRRC2C	Protein PRRC2C [OS = *Homo sapiens*]	0.42	0.0045	nan
MTDH	Protein LYRIC [OS = *Homo sapiens*]	0.42	0.0014	nucleic acid binding activity; other molecular function
RBM14	RNA-binding protein 14 [OS = *Homo sapiens*]	0.42	0.0024	other molecular function
RPS4X	40S ribosomal protein S4, X isoform [OS = *Homo sapiens*]	0.41	0.0025	nucleic acid binding activity; other molecular function
PDLIM7	PDZ and LIM domain protein 7 [OS = *Homo sapiens*]	0.4	0.0022	cytoskeletal activity; other molecular function
H2afy; H2AFY; MACROH2A1	Core histone macro-H2A.1 [OS = *Homo sapiens*]	0.39	0.0045	enzyme regulator activity; nucleic acid binding activity; other molecular function
YTHDF1	YTH domain-containing family protein 1 [OS = *Homo sapiens*]	0.39	0.0003	translation activity; nucleic acid binding activity
UBAP2L	Ubiquitin-associated protein 2-like [OS = *Homo sapiens*]	0.38	0.0016	nan
RBM28	RNA-binding protein 28 [OS = *Homo sapiens*]	0.37	0.0009	nan
SRRM2	Serine/arginine repetitive matrix protein 2 [OS = *Homo sapiens*]	0.37	0.0052	nucleic acid binding activity; other molecular function
TAGLN2	Transgelin-2 [OS = *Homo sapiens*]	0.37	0.0002	cytoskeletal activity
RPS16	40S ribosomal protein S16 [OS = *Homo sapiens*]	0.36	0.0002	nucleic acid binding activity; other molecular function
SMU1	WD40 repeat-containing protein SMU1 [OS = *Homo sapiens*]	0.36	0.0041	nan
KIF2C	Kinesin-like protein KIF2C [OS = *Homo sapiens*]	0.36	0.0071	cytoskeletal activity; nucleic acid binding activity; other molecular function
NSA2	Ribosome biogenesis protein NSA2 homolog [OS = *Homo sapiens*]	0.35	0.0051	nan
MAP7	Ensconsin [OS = *Homo sapiens*]	0.35	0.0005	signal transduction activity or receptor binding; other molecular function
DNTTIP2	Deoxynucleotidyltransferase terminal-interacting protein 2 [OS = *Homo sapiens*]	0.35	0.0001	nan
HP1BP3	Heterochromatin protein 1-binding protein 3 [OS = *Homo sapiens*]	0.34	0.0045	nucleic acid binding activity; other molecular function
KPNA3	Importin subunit alpha-4 [OS = *Homo sapiens*]	0.34	0.0096	other molecular function
UQCRFS1	Cytochrome b-c1 complex subunit Rieske, mitochondrial [OS = *Homo sapiens*]	0.32	0.0085	transporter activity; other molecular function
LRRC47	Leucine-rich repeat-containing protein 47 [OS = *Homo sapiens*]	0.31	0.0033	other molecular function
RPS11	40S ribosomal protein S11 [OS = *Homo sapiens*]	0.3	0.0004	nucleic acid binding activity; other molecular function
RPL23	60S ribosomal protein L23 [OS = *Homo sapiens*]	0.3	0.0013	enzyme regulator activity; nucleic acid binding activity; other molecular function
nan	Probable ATP-dependent RNA helicase DDX52 [OS = *Homo sapiens*]	0.3	0.0057	nan
EXOSC6	Exosome complex component MTR3 [OS = *Homo sapiens*]	0.3	0.006	nan
MAP1S	Microtubule-associated protein 1S [OS = *Homo sapiens*]	0.3	0.0086	cytoskeletal activity; nucleic acid binding activity; other molecular function
HMGB2	High mobility group protein B2 [OS = *Homo sapiens*]	0.29	0.0011	signal transduction activity or receptor binding; nucleic acid binding activity; other molecular function
TCOF1	Treacle protein [OS = *Homo sapiens*]	0.28	0.004	other molecular function
RPL13	60S ribosomal protein L13 [OS = *Homo sapiens*]	0.28	0.0097	other molecular function
MKI67	Proliferation marker protein Ki-67 [OS = *Homo sapiens*]	0.28	0.0008	nucleic acid binding activity; other molecular function
RPS14	40S ribosomal protein S14 [OS = *Homo sapiens*]	0.26	0.0014	nucleic acid binding activity; other molecular function
YY1	Transcriptional repressor protein YY1 [OS = *Homo sapiens*]	0.26	0.0066	nucleic acid binding activity; other molecular function
RPL37A	60S ribosomal protein L37a [OS = *Homo sapiens*]	0.25	0.0051	other molecular function
SAFB	Scaffold attachment factor B1 [OS = *Homo sapiens*]	0.25	0.0088	nucleic acid binding activity; other molecular function
DDX49	Probable ATP-dependent RNA helicase DDX49 [OS = *Homo sapiens*]	0.24	0.0045	other molecular function
CDC27	Cell division cycle protein 27 homolog [OS = *Homo sapiens*]	0.24	0.0036	other molecular function
CBX8	Chromobox protein homolog 8 [OS = *Homo sapiens*]	0.24	0.0099	enzyme regulator activity; nucleic acid binding activity; other molecular function
RPS24	40S ribosomal protein S24 [OS = *Homo sapiens*]	0.24	0.0099	other molecular function
CNN2	Calponin-2 [OS = *Homo sapiens*]	0.23	0.0067	cytoskeletal activity; other molecular function
TAF15	TATA-binding protein-associated factor 2N [OS = *Homo sapiens*]	0.23	0.0058	nucleic acid binding activity; other molecular function
MARK3	MAP/microtubule affinity-regulating kinase 3 [OS = *Homo sapiens*]	0.22	0.0017	cytoskeletal activity; kinase activity; other molecular function
TRIM32	E3 ubiquitin-protein ligase TRIM32 [OS = *Homo sapiens*]	0.22	0.0045	cytoskeletal activity; nucleic acid binding activity; other molecular function
MAP4	Microtubule-associated protein 4 [OS = *Homo sapiens*]	0.22	0.0023	cytoskeletal activity; other molecular function
NOLC1	Nucleolar and coiled-body phosphoprotein 1 [OS = *Homo sapiens*]	0.2	0.002	nucleic acid binding activity; other molecular function
MDC1	Mediator of DNA damage checkpoint protein 1 [OS = *Homo sapiens*]	0.2	0.0038	nan
LARP1	La-related protein 1 [OS = *Homo sapiens*]	0.19	0.0008	translation activity; nucleic acid binding activity; other molecular function
PRKAR1A	cAMP-dependent protein kinase type I-alpha regulatory subunit [OS = *Homo sapiens*]	0.19	0.0031	enzyme regulator activity; other molecular function
RPS8	40S ribosomal protein S8 [OS = *Homo sapiens*]	0.18	0.0059	other molecular function
RPL26	60S ribosomal protein L26 [OS = *Homo sapiens*]	0.18	0.0096	nucleic acid binding activity; other molecular function
MINK1	Misshapen-like kinase 1 [OS = *Homo sapiens*]	0.17	0.0056	kinase activity; other molecular function
H1-1	Histone H1.1 [OS = *Homo sapiens*]	0.16	0.0002	nucleic acid binding activity; other molecular function
RRP1B	Ribosomal RNA processing protein 1 homolog B [OS = *Homo sapiens*]	0.16	0.0073	other molecular function
SRRM1	Serine/arginine repetitive matrix protein 1 [OS = *Homo sapiens*]	0.16	0.0001	nucleic acid binding activity
HNRNPA3	Heterogeneous nuclear ribonucleoprotein A3 [OS = *Homo sapiens*]	0.16	0.0018	transporter activity; nucleic acid binding activity
PPME1	Protein phosphatase methylesterase 1 [OS = *Homo sapiens*]	0.15	0.0	enzyme regulator activity; nucleic acid binding activity; other molecular function
RPL32	60S ribosomal protein L32 [OS = *Homo sapiens*]	0.15	0.0004	other molecular function
UTP18	U3 small nucleolar RNA-associated protein 18 homolog [OS = *Homo sapiens*]	0.14	0.0062	nan
RRM2	Ribonucleoside-diphosphate reductase subunit M2 [OS = *Homo sapiens*]	0.14	0.0029	other molecular function
H1-2	Histone H1.2 [OS = *Homo sapiens*]	0.14	0.0067	nucleic acid binding activity; other molecular function
RPL36AL	60S ribosomal protein L36a-like [OS = *Homo sapiens*]	0.14	0.0081	other molecular function
RPS29	40S ribosomal protein S29 [OS = *Homo sapiens*]	0.12	0.0013	other molecular function
RRN3	RNA polymerase I-specific transcription initiation factor RRN3 [OS = *Homo sapiens*]	0.11	0.0005	nucleic acid binding activity; other molecular function
RFC4	Replication factor C subunit 4 [OS = *Homo sapiens*]	0.1	0.0031	nucleic acid binding activity; other molecular function
BAZ1B	Tyrosine-protein kinase BAZ1B [OS = *Homo sapiens*]	0.09	0.0051	kinase activity; other molecular function
EIF4H	Eukaryotic translation initiation factor 4H [OS = *Homo sapiens*]	0.08	0.0008	nucleic acid binding activity
H1-10	Histone H1.10 [OS = *Homo sapiens*]	0.08	0.0045	nucleic acid binding activity; other molecular function
H1-4	Histone H1.4 [OS = *Homo sapiens*]	0.07	0.006	nucleic acid binding activity; other molecular function
RPL39	60S ribosomal protein L39 [OS = *Homo sapiens*]	0.06	0.0096	nucleic acid binding activity; other molecular function
LAD1	Ladinin-1 [OS = *Homo sapiens*]	0.05	0.0002	other molecular function
HMGA1	High mobility group protein HMG-I/HMG-Y [OS = *Homo sapiens*]	0.01	0.0025	signal transduction activity or receptor binding; nucleic acid binding activity; other molecular function
HMGA2	High mobility group protein HMGI-C [OS = *Homo sapiens*]	0.01	0.0081	nucleic acid binding activity; other molecular function
